# Roles of SPARC in urothelial carcinogenesis, progression and metastasis

**DOI:** 10.18632/oncotarget.11590

**Published:** 2016-08-24

**Authors:** Neveen Said

**Affiliations:** ^1^ Department of Cancer Biology, Wake Forest University Health Sciences, Winston Salem, NC, USA

**Keywords:** SPARC, urinary bladder, carcinogenesis, cell cycle, inflammation

## Abstract

Secreted Protein Acidic and Rich in Cysteine (SPARC) is a matricellular glycoprotein that is implicated in myriad physiological and pathological conditions characterized by extensive remodeling and plasticity. The functions and disease association of SPARC in cancer is being increasingly appreciated as it plays multi-faceted contextual roles depending on the cancer type, cell of origin and the unique cancer milieu at both primary and metastatic sites. Herein we will review our current knowledge of the role of SPARC in the multistep cascades of urinary bladder carcinogenesis, progression and metastasis from preclinical models and clinical data and shine the light on its prognostic and therapeutic potentials.

## STRUCTURE AND BIOCHEMICAL PROPERTIES OF SPARC

**Figure 1:**
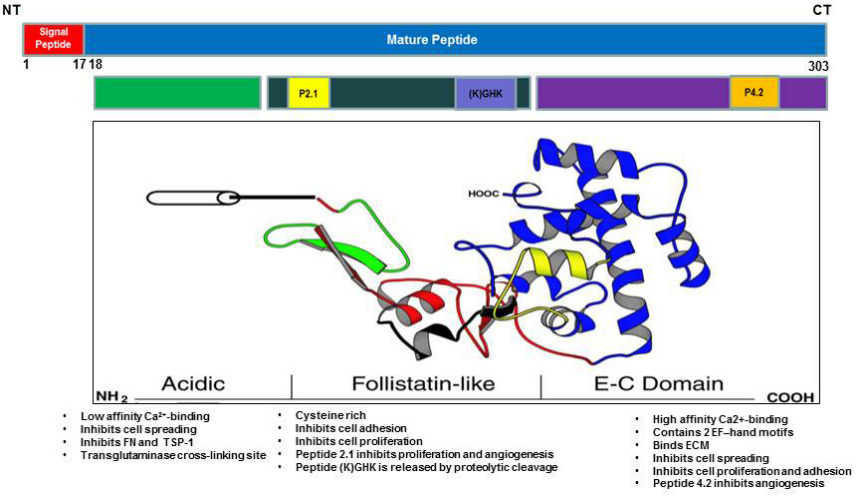
Schematic illustration of SPARC molecule showing the functional domains

SPARC is a matricellular glycoprotein that has been associated with extensive tissue remodeling and tumorigenesis. *SPARC* gene was initially discovered as a bone matrix and an endothelial basement membrane protein (hence the names osteonectin/BM40). SPARC protein is encoded by a single gene in human chromosome 5q31.1 and mouse chromosome 11 [1–3]. Mature SPARC protein has 286 amino acids with three distinct functional domains, including an N-terminus acidic domain (NT), follistatin-like domain (FS) and C-terminus domain (EC). The NT domain, spans the first 52 amino acids, is highly acidic and binds hydroxyapatite with low affinity Ca^2+^ binding (5-8 Ca^2+^) [3, 4]. The FS comprises the next 85 amino acids and contains several internal disulfide bonds and N-glycosylation site. The EC domain is 149 amino acids and contains two EF-hand motifs that bind calcium with high affinity and is comprised almost entirely of β-helices (Figure 1).

The biological functions of SPARC were depicted from the phenotypes of *Sparc*-deficient mice (*SP^−/−^*) and were related to defects of fibroblast and myeloid differentiation and plasticity as cataract formation, osteopenia, decreased size and tensile strength of collagen fibers, and increased deposition of adipose tissue [5, 6]. More biological functions evolved when these mice were challenged as accelerated wound and defective organ healing after injury, increased angiogenesis as well as accelerated growth of implanted syngeneic subcutaneous (SC) tumors. All were initially attributed to increased proliferation and angiogenesis with defective matrix assembly and encapsulation [7–19]. Unique contextual biological functions of SPARC were identified from disease/organ-specific models. In cancer, our knowledge of the contextual expression and functions of SPARC was furthered by orthotopic as well as autochthonous (oncogene-driven and carcinogen-induced) models of cancer together with xenografts of human cancer cells in immuno-deficient mice and *in vitro* 2D and 3D cell culture systems [7, 19–25]. In addition, SPARC is not directly implicated in the cellular transformation and cancer initiation as spontaneous cancers do not develop in mice with germline deletion of SPARC. However, SPARC is significantly implicated in the pathobiology of many cancers where it influences tumor-stromal interactions in both autocrine and paracrine manners modulating tumor progression and response to therapy (summarized in [19]).

The role of SPARC in tumor development and metastasis is contextual. It depends not only on cancer type, but on whether the molecule is produced by cancer cells or surrounding stromal cells, its subcellular localization in a given cell type, the composition of the ECM, as well as on its interactions with the biologically active molecules in a given tumor milieu [19, 23, 26]. Stromal (host) SPARC regulates ECM deposition and modulates tumor growth and progression [7, 8, 19, 22, 23, 27, 28]. SPARC normalizes the tumor microenvironment (TME) through anti-inflammatory properties and regulation of integrin-growth factor receptor interactions [7, 19, 22–24, 26] or through a regulation of MMPs release [27, 29, 30]. SPARC exerts an autocrine and a paracrine inhibition of tumor cell proliferation [7, 19, 22–24, 28, 31–33] evident in cell cycle arrest at G1/S phase [19, 28]. In addition, tumor SPARC is subject to epigenetic silencing through promoter methylation in many cancers [26, 28, 34–41]. Herein, we review the current knowledge of the role and association of SPARC in bladder cancer.

## BLADDER CANCER

**Figure 2:**
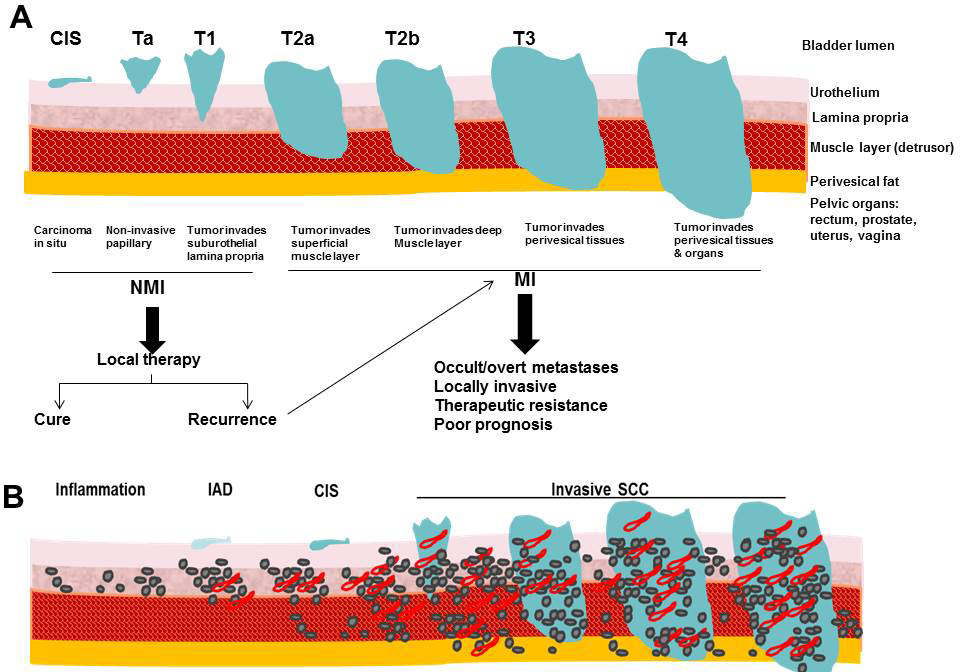
Comparative illustration of **A.** Staging of human bladder cancer according to the Tumor-Node-Metastasis (TNM) system, **B.** pathological stages in carcinogen-induced murine model of bladder cancer.

Bladder cancer is the most common malignancy affecting the urinary system with estimated 76,960 new cases and projected 16,390 deaths in 2016 in the United States. This represents ∼4% increase in new cases and 2.4% increase in mortality from 2015 [42]. Patients with bladder cancer present at or after 5^th^ decade of life with male: female ratio of 3:1 [42, 43]. Environmental risk factors include tobacco smoking, occupational exposure to aromatic amines and polycyclic hydrocarbons, consumption of arsenic-contaminated water, chronic infections, ionizing radiations and therapeutic abuse of phenacetin-containing analgesics. Tobacco smoking is the major environmental risk factor. It is estimated that > 50% of cases of bladder cancer can be attributed to smoking [44]. The most common form of bladder cancer is urothelial carcinoma (UC), formerly known as transitional cell carcinoma that arises from the mucosal lining of the bladder, and frequently occurs as a multifocal disease involving several simultaneous tumors scattered over the urothelium (Figure 2). Urothelial carcinomas often exhibit elements of squamous or glandular differentiation, which is more common in high-grade and high-stage lesions [45]. Other less frequent pathological subtypes include squamous cell carcinoma, adenocarcinoma, and small cell carcinoma [45]. Most patients present with non-muscle invasive disease (NMI) that is treated with bladder preserving endoscopic resection and adjuvant intra-vesical therapies. However, recurrence is high with 50% chances to progress to muscle invasive (MI) disease (reviewed in [46, 47]). Patients who present with MI disease are treated best with aggressive local therapy (e.g. radical cystectomy, radiotherapy) and/or systemic chemotherapy [47]. Half of the patients with MI disease or progressing to it from NMI disease harbor occult metastases with significantly poor prognosis and survival [47]. Because of its protracted natural history, bladder cancer is among the most expensive malignancies to treat from diagnosis to death. Treatments have not advanced in the past 30 years and there is no approved second line of therapy [46, 47], so there is a crucial unmet need for novel therapies, especially for advanced disease.

Numerous genetic and epigenetic alterations were implicated in tumorigenesis and progression of bladder cancer. The Cancer Genome Atlas (TCGA) data [48] revealed statistically significant recurrent mutations in 32 genes, including multiple genes involved in cell-cycle regulation, chromatin regulation, and kinase signaling pathways*.* However because this disease, like other cancers, consists of biologically heterogeneous cell populations, selection pressures inferred from the microenvironment, favor development of subpopulations of cells with different dormant, invasive and metastatic abilities. In this respect, SPARC represents an interesting molecule as it exerts distinct roles on cancer cells and tissue-specific stromal cells and modulates the interactions of cancer cells with the cellular and acellular components of the surrounding tumor microenvironment (TME).

### The expression of SPARC in normal urinary bladder

In the normal bladders, SPARC protein is expressed in basal and luminal surfaces of normal murine and human urothelia and is secreted by primary urothelial cultures [19, 49]. SPARC exerted anti-proliferative and de-adhesive effects on cultured normal human and murine urothelial cells *in vitro* [50]. SPARC is dispensable for bladder development and function as evidenced by the normal development and function of mice with germline deletion of SPARC. SPARC protein is also expressed in the sub-urothelial stroma [19]. Further studies of the main two stromal components, fibroblasts and macrophages, indicated that both cell types express and secrete SPARC that is required for their differentiation and phenotypic commitment in response to micro-environmental cues [19].

### SPARC expression in human bladder cancer is associated with advancer stage and poor outcome

**Figure 3:**
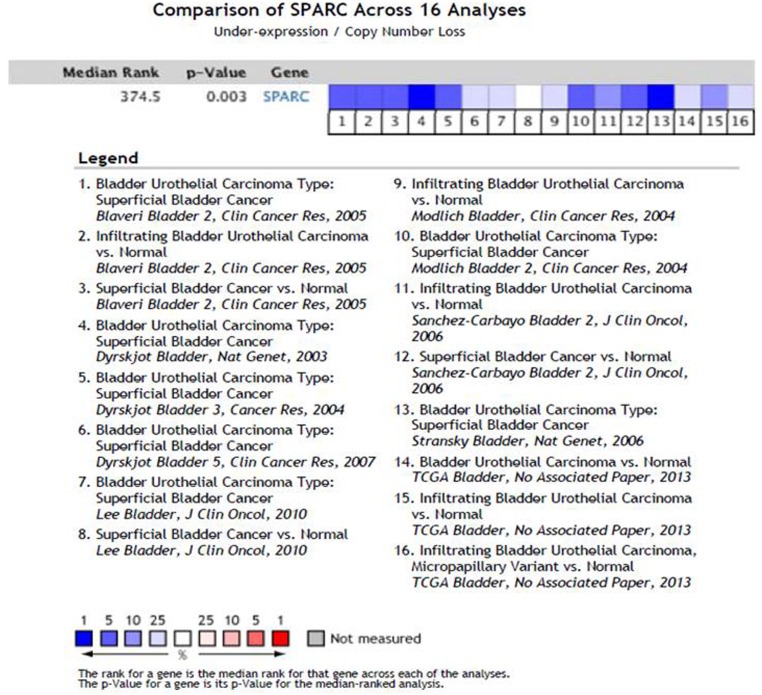
Comparison of SPARC expression/copy number across 16 Analyses of gene expression/copy number analysis in human bladder cancer

In human bladder cancer tissue microarrays (TMA) that comprised 192 patients’, SPARC protein expression significantly decreased in MI compared to NMI disease [19]. In NMI disease, SPARC is expressed in the cancerous urothelium and adjacent stroma. In contrast, in MI disease, the expression of SPARC exhibited distinctive compartmentalization with decrease in the frequency and intensity of staining in the cancerous tissue while positive staining was mainly observed in the tumor associated stroma. SPARC expression inversely correlated with disease-specific survival (DSS). However, there was no relationship between the intensity and/or frequency of stromal SPARC expression and DSS. Consistently, RNA and copy number analysis data curated from oncomine database (www.oncomine.org) indicated down-regulation/low copy number of SPARC transcript in 15/16 studies (Figure 3).

### Effect of SPARC on carcinogen-induced urothelial transformation

SPARC transcript and protein expression were reported to be significantly downregulated by carcinogenic heavy metals during malignant transformation of UROtsa cell line [51]. The clinically-related studies appeared descriptive and represent snap shots of archival tumor samples. Therefore, it was imperative to develop multiple preclinical models to comprehensively investigate the roles of SPARC in multi-step carcinogenesis and metastasis cascades [19]. The relevance of SPARC in the hallmarks of bladder cancer was determined using a carcinogen-induced model providing a tobacco metabolite and chemical carcinogen *N*-nitrosobutyl(4-hydroxybutyl)amine (BBN) to *SP^−/−^* and *SP^+/+^* mice for ∼ 40 weeks. This model recapitulated human bladder cancer that develops after prolonged exposure to chemical carcinogens and allowed the study the pre-neoplastic lesions that are not clinically encountered [19] (Figure 2). This model in tandem with preclinical models using human cell lines *in vitro* and in xenografts allowed study of the effect of SPARC on the hallmarks of cancer [52] that encompass fundamental biological capabilities acquired during cell transformation and cancer development including sustained proliferation, evasion of growth suppression, death resistance, replicative immortality, induced angiogenesis, and initiation of invasion and metastasis.

### SPARC restrains cell proliferation and cell cycle progression

Cellular transformation and tumor progression require escape from proliferative suppression and the tightly controlled cell cycle by the G1/S cell cycle checkpoint. SPARC-deficient urothelial lesions exhibited a significant progressive increase in proliferation compared to SPARC-proficient lesions with significantly increasing higher levels of cyclins A1, D1 and E2 that drive cell cycle progression and were positively correlated with the presence, invasion, progression and metastasis of human bladder cancer [53, 54]. Paradoxically, the expression of cell cycle inhibitors p21*^CIP/WAF1^* and p27*^KIP1^* significantly and progressively decreased in SPARC-deficient compared to SPARC-proficient urothelial lesions; a finding that phenocopies their perturbed expression in human urothelial cancer [55–65] and further supports the tumor suppressor effect of SPARC in part through inhibition of cell cycle progression at G1/S.

Interestingly, the expression of SPARC protein and transcript were significantly downregulated in tumorigenic bladder cancer cell line T24T compared to its non-tumorigenic isogenic line T24 [19, 66]. SPARC expression in human bladder cancer cell lines inversely correlated with their proliferation rate, restrained cell cycle progression through slowing G1/S cell cycle proteins, cyclins A1, D1 and E2 with increase of their inhibitors p21*^CIP/WAF1^* and p27^KIP1^. Of interest is that in human bladder cancer, cyclin D1 (*CCND1*) is amplified in 20%, whereas inactivating mutations, hemizygous and homozygous deletions of the tumor suppressor *CDKN1A* gene that encodes p21*^CIP/WAF1^* have been recently reported with higher frequencies of deletion in muscle invasive (MI) disease [44]. The inhibition of activation of cJun/AP1 by SPARC [19] suggests that inhibition of cell cycle deregulation is mediated in part through inhibition of the upstream transcription factor. In addition, TCGA data [48] revealed the enrichment of signaling pathways that were inhibited by SPARC in ovarian cancer [7, 23, 24] as phosphatidylinositol-3-OH kinase/AKT/mTOR pathway and the RTK/MAPK pathway suggesting potential inhibitory effect in bladder cancer milieu.

### Anti-oxidant and anti-inflammatory

**Figure 4:**
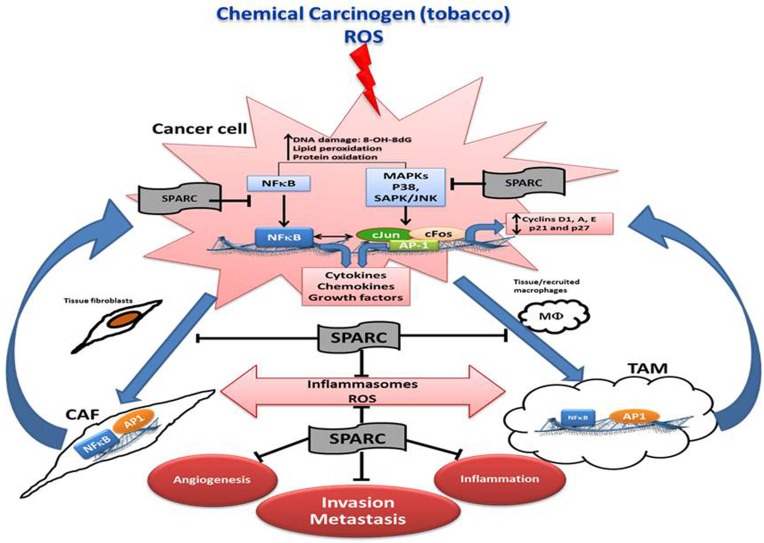
Schematic illustration summarizing the effects of SPARC on the interactions of cancer cells, and stromal cells in the multistep carcinogenesis cascade

In carcinogen-induced urothelial lesions, SPARC inhibited the progressive generation of ROS and markers of DNA, protein and lipid oxidative damage; a scenario that was significantly augmented in *SP^−/−^* urothelial lesions. SPARC-proficient (*SP^+/+^*) lesions exhibited significantly decreased activation of the downstream signaling cascades that converged in the activation of AP-1 and NFκB, the major orchestrators of inflammation, carcinogenesis, invasiveness and metastasis [19, 67–69]. High levels of ROS (H_2_O_2_) are generated by urothelial cancer cells compared to normal urothelial cells implying that ROS are generated upon carcinogen exposure and from enhanced metabolism of the rapidly proliferating transformed cells [19]. This explains, in part, the significantly higher levels of ROS in the rapidly proliferating *SP^−/−^* urothelial cells. High ROS concentrations are generated by cancer cells and by the surrounding juxta-tumoral stromal cells mainly TAMs and CAFs [19, 70] (Figure 4).

The increased ROS in the evolving urothelial cancer milieu was manifested by progressive increased levels of 8-hydroxy-2′ -deoxyguanosine (8-OHdG) that contributes to genome stability and promotes carcinogenesis [71]. Chronic oxidative stress through DNA damage has been shown to induce to double strand breaks (DSBs) with complex DNA ends. Repairing such complex DSBs with non-homologous end joining (NHEJ) may be critical for the production of spontaneous mutations and inflammation-related cancers [72]. Mitochondrial DNA and nuclear DNA undergo several alterations that may result in mutation accumulation and genomic instability [72, 73]. In lieu of these reports we can speculate that SPARC, through its anti-oxidant and anti-inflammatory effects may exert a protective role against carcinogen- and ROS-induced genome instability and mutations.

Oncogenic transformation induces metabolic reprogramming of cancer cells with increased glucose and glutamine metabolism and, consequently, increased proliferation and ROS production [73–75]. Oncogenic transcription factors as NFκB, and AP-1 are activated by ROS and in synergy with the specific signaling from their downstream target genes may further perturbation of cellular energy and metabolism [73–75]. The rapidly proliferating cells in the absence of SPARC along with activation of pro-oncogenes and loss of tumor suppressors generate increased levels of ROS, a manifestation of altered cellular metabolism, may induce aerobic glycolysis or Warburg effect [49].

The kinetics of SPARC protein expression during differentiation of primary fibroblasts and macrophages revealed that it increases during early differentiation, then decreases to basal levels in macrophages but remained steady in differentiated fibroblasts. Mechanistic studies using heterotypic co-cultures of normal and cancerous urothelial cells with normal and tumor associated stromal cells indicated that SPARC inhibited the acquisition inflammatory secretory phenotype of tumor associated macrophages (TAMs) and cancer associated fibroblasts (CAFs) through inhibition of the activation of NFκB and AP-1 with subsequent decrease in their secreted cytokines and cancer cell invasiveness. These findings suggested that SPARC markedly inhibited the inflammatory feed-forward loop that is reciprocated and maintained among cancer cells, TAMs, and CAFs through secreted inflammasomes (ROS, bioactive lipids, cytokines, chemokines and growth factors) that act as a double-edged sword in the tumor microenvironment. On the one hand, they sustain cancer cell proliferation, invasiveness, angiogenesis, and metastasis. On the other hand, they play a critical role in recruitment and differentiation of stromal cells.

In addition, the inflammatory TME in low/absence of SPARC can contribute to immune suppression enabling tumor cells to evade the immune system and disseminate [76]. Therefore, the kinetics of SPARC expression in cancerous and stromal cells in human and murine urothelial cancer implicate SPARC in the intricate tightly-regulated programs of cellular recruitment, proliferation, differentiation/de-differentiation of stromal cells [5, 6, 10, 19, 77, 78]. The differential compartmentalization of SPARC may represent a state of aberrant homeostasis with increased inflammation that might be directly involved in urothelial cell transformation through the persistent release of inflammatory mediators and ROS, ancillary to tumor growth and metastasis (Figure 4).

### Inhibition of angiogenesis

Tumor angiogenesis is one of the hallmarks of cancer [52]. Initial growth of transformed cells and tumor mass increases the demands for oxygen from blood, thus tumor tissue becomes hypoxic. In turn, hypoxia produces ROS which activate potent angiogenic hypoxia inducible factor (HIF-1α) to increase the transcription of angiogenic growth factors [79]. Angiogenesis is stimulated by growth factors such as VEGF, FGF as well as pro-inflammatory mediators enriched in the TME which induce the proliferation and migration of endothelial cells followed by assembly into patent blood vessels [79, 80]. The anti-angiogenic effects of SPARC have been long recognized as evidenced by its inhibitory effect on endothelial cell proliferation and migration *in vitro* and *in vivo* by increased angiogenesis and tumor growth in syngeneic tumors implanted in *SP^−/−^* mice and paradoxically, decreased angiogenesis and growth of human tumor xenografts expressing SPARC [7, 9, 19, 21–25, 81–83]. In urothelial cancer, the anti-angiogenic effect of SPARC is attributed to its anti-oxidant, anti-inflammatory effects with subsequent inhibition of angiogenesis at multiple levels [19]. SPARC significantly suppressed carcinogen-induced ROS generation and inflammation while restraining cell cycle progression in cancer cells, thus limiting the increasing demands of the growing tumor cells to new blood vessels. Through modulation of cancer cells’ interactions with stromal cells, SPARC inhibited phenotypic commitment of macrophages and fibroblasts into inflammatory TAM and CAF phenotype. Consequently, the pro-angiogenic factors in the tumor milieu significantly decreased, with subsequent inhibition of endothelial cell recruitments, angiogenesis and vasculogenesis. Furthermore, the observation that SPARC-deficient autochthonous urothelial cancers and in syngeneic tumors growing in *SP^−/−^* mice exhibited increased vascularity macroscopically with the increased mean vascular density and area (number and size of blood vessels) strongly implicates a direct effect of host and/or tumor SPARC on endothelial cells in the multistep cascade of tumor angiogenesis and vasculogenesis.

### Inhibition of invasion and metastasis

**Figure 5:**
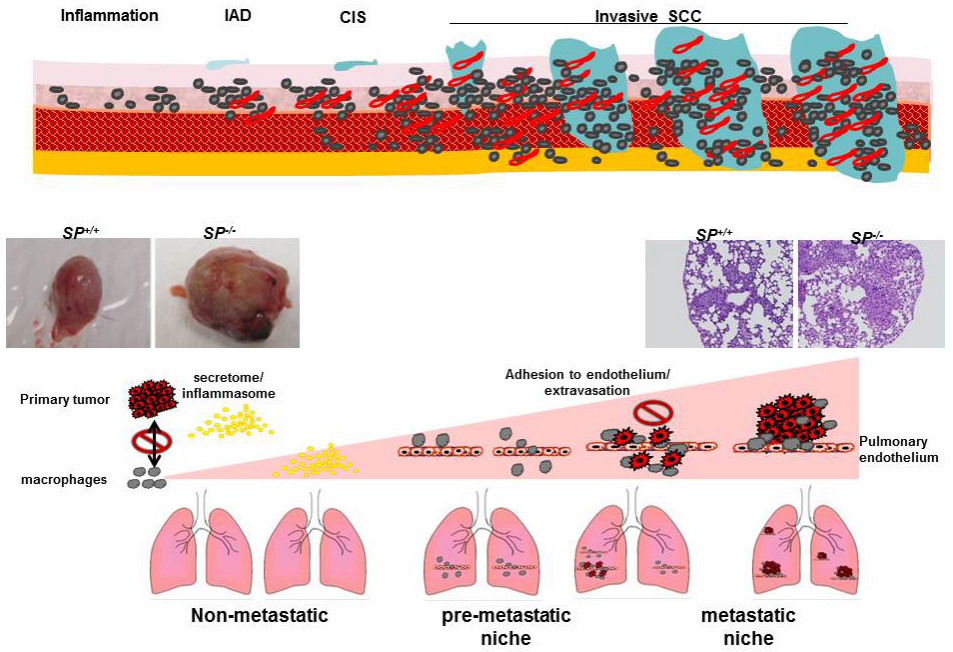
A view of the effect of SPARC on the multistep metastasis cascade and preconditioning pf the metastatic niche

The process of metastasis is defined by distinct steps involving local invasion, intravasation into adjacent blood and lymphatic vessels, transit through circulation and evasion of host immune systems, progressive preconditioning of the prospective metastatic sites, extravasation into the parenchyma of metastatic site, and colonization and formation of micro-metastases, followed by proliferation and progression to macro-metastases. This process is largely inefficient due to the many obstacles tumor cells must overcome to successfully metastasize. In this respect SPARC exerts an anti-metastatic effect at multiple levels. In the carcinogen-induced bladder cancer, *SP^−/−^* mice not only exhibited accelerated growth that involved the full bladder wall with invasion of the muscle layer and peri-vesical tissues, but also exhibited early onset metastasis and significantly more metastases [19]. Primary *SP^−/−^* tumors exhibited significantly higher pro-inflammatory and pro-invasive mediators and were more vascular than *SP^+/+^* tumors. Pairwise examination of matching lungs and bladder tissues with pre-neoplastic and neoplastic lesions revealed progressive increase in the levels of pro-inflammatory mediators with significantly higher levels in *SP^−/−^* compared with *SP^+/+^* lungs as a function of disease progression. In addition, macrophage infiltration was significantly higher in *SP^−/−^* compared with the *SP^+/+^* lung metastases. These data suggest that the anti-metastatic effect of SPARC is mediated in part through its anti-inflammatory effect on the primary tumor, suppressing the pre-conditioning of the pre-metastatic lung tissues, “pre-metastatic niche” [19, 84–87] (Figure 5).

The contribution of cancer cell- *vs.* stromal cell- SPARC in bladder cancer metastasis was dissected using multiple independent approaches. Using a syngeneic model of spontaneous metastasis in which SPARC-proficient MB49 cells were injected SC in *SP^−/−^* and *SP^+/+^* mice, host-SPARC not only inhibited the *in vivo* growth, invasiveness, angiogenesis and inflammation of primary tumors, but it also inhibited spontaneous lung metastasis through inhibition of the pre-conditioning of the inflammatory pre-metastatic and metastatic lung niche [19] (Figure 5).

The role of tumor-SPARC in lung colonization was further investigated in a human experimental metastasis model, injecting human bladder cancer cells genetically modified for SPARC expression into nude mice [19]. SPARC expression inversely correlated with the incidence and multiplicity of lung metastasis. Of note, bladder cancer cells depleted of SPARC exhibited a dramatic decrease in early lung colonization implicating a counter-adhesive effect of SPARC inhibiting tumor cell adhesion to pulmonary endothelial cells and early metastatic colonization.

## CONCLUSIONS AND PERSPECTIVE

**Figure 6:**
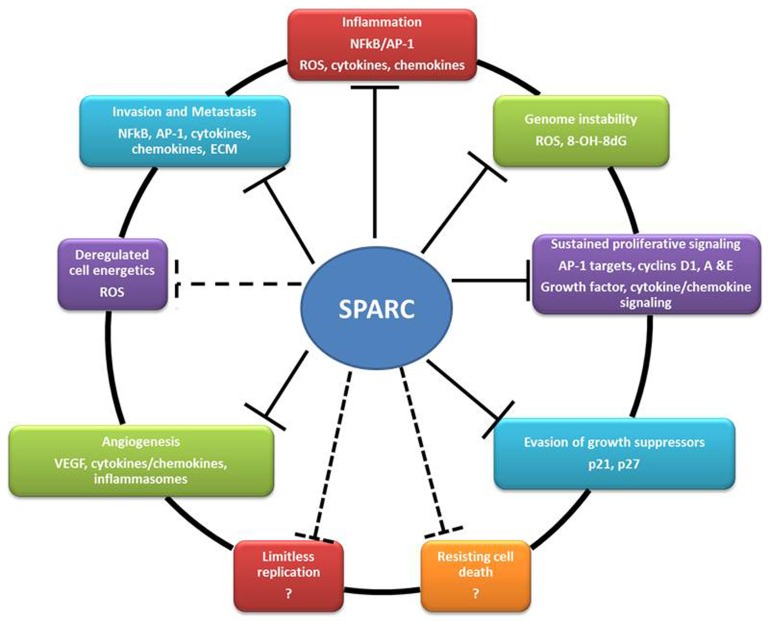
Summary of the reported effects of SPARC on the hallmarks of cancer (solid lines) and the potential effects (dashed lines)

A large body of research over the last three decades has established SPARC as an ECM protein that plays a substantial role in tissue homeostasis in physiological and pathological contexts characterized by proliferation, differentiation and plasticity of mesenchymal, myeloid, neuronal as well as endothelial and epithelial cells. The role of SPARC in tumorigenesis has been recently recognized and new functions of SPARC in disease, organ and cell-specific contexts are being unveiled. In bladder cancer, our reports strongly suggest a tumor suppressor effect of SPARC modulating the putative hallmarks of cancer (Figure 6). Collectively, findings from our published reports strongly suggest a SPARC as a potential prognostic biomarker in bladder cancer. The identification of the molecular signature associated with SPARC gene/protein expression could serve as prognostic biomarkers to stratify patients who might develop invasive, metastatic, indolent or recurrent disease. In addition to prognostication, in the era of precision and personalized medicine, it is imperative that future investigations develop and test specific pharmacological agents that target SPARC and/or its regulating/regulated signaling pathways in preclinical and clinical settings.
